# Supplementary material for: A fault-intrusion-tolerant system and deadline-aware algorithm for scheduling scientific workflow in the cloud
Source: PeerJ Comput Sci. 2021 Nov 2;7:e747. doi: 10.7717/peerj-cs.747 (PMC8576554; doi:10.7717/peerj-cs.747)
Supplement: Supplemental Information 6 [file peerj-cs-07-747-s006.zip › Myproject1.1/docs/org/cloudbus/cloudsim/examples/power/random/package-summary.html]

org.cloudbus.cloudsim.examples.power.random


JavaScript is disabled on your browser.


- Overview
- Package
- Class
- Use
- Tree
- Deprecated
- Index
- Help

- Prev Package
- Next Package

- Frames
- No Frames

- All Classes

# Package org.cloudbus.cloudsim.examples.power.random

- Class Summary

  | Class | Description |
  | --- | --- |
  |  |  |
  | --- | --- |
  | Dvfs | A simulation of a heterogeneous power aware data center that only applied DVFS, but no dynamic optimization of the VM allocation. |
  | IqrMc | A simulation of a heterogeneous power aware data center that applies the Inter Quartile Range (IQR) VM allocation policy and Maximum Correlation (MC) VM selection policy. |
  | IqrMmt | A simulation of a heterogeneous power aware data center that applies the Inter Quartile Range (IQR) VM allocation policy and Minimum Migration Time (MMT) VM selection policy. |
  | IqrMu | A simulation of a heterogeneous power aware data center that applies the Inter Quartile Range (IQR) VM allocation policy and Minimum Utilization (MU) VM selection policy. |
  | IqrRs | A simulation of a heterogeneous power aware data center that applies the Inter Quartile Range (IQR) VM allocation policy and Random Selection (RS) VM selection policy. |
  | LrMc | A simulation of a heterogeneous power aware data center that applies the Local Regression (LR) VM allocation policy and Maximum Correlation (MC) VM selection policy. |
  | LrMmt | A simulation of a heterogeneous power aware data center that applies the Local Regression (LR) VM allocation policy and Minimum Migration Time (MMT) VM selection policy. |
  | LrMu | A simulation of a heterogeneous power aware data center that applies the Local Regression (LR) VM allocation policy and Minimum Utilization (MU) VM selection policy. |
  | LrrMc | A simulation of a heterogeneous power aware data center that applies the Local Regression Robust (LRR) VM allocation policy and Maximum Correlation (MC) VM selection policy. |
  | LrrMmt | A simulation of a heterogeneous power aware data center that applies the Local Regression Robust (LRR) VM allocation policy and Minimum Migration Time (MMT) VM selection policy. |
  | LrrMu | A simulation of a heterogeneous power aware data center that applies the Local Regression Robust (LRR) VM allocation policy and Minimum Utilization (MU) VM selection policy. |
  | LrrRs | A simulation of a heterogeneous power aware data center that applies the Local Regression Robust (LRR) VM allocation policy and Random Selection (RS) VM selection policy. |
  | LrRs | A simulation of a heterogeneous power aware data center that applies the Local Regression (LR) VM allocation policy and Random Selection (RS) VM selection policy. |
  | MadMc | A simulation of a heterogeneous power aware data center that applies the Median Absolute Deviation (MAD) VM allocation policy and Maximum Correlation (MC) VM selection policy. |
  | MadMmt | A simulation of a heterogeneous power aware data center that applies the Median Absolute Deviation (MAD) VM allocation policy and Minimum Migration Time (MMT) VM selection policy. |
  | MadMu | A simulation of a heterogeneous power aware data center that applies the Median Absolute Deviation (MAD) VM allocation policy and Minimum Utilization (MU) VM selection policy. |
  | MadRs | A simulation of a heterogeneous power aware data center that applies the Median Absolute Deviation (MAD) VM allocation policy and Random Selection (RS) VM selection policy. |
  | NonPowerAware | A simulation of a heterogeneous non-power aware data center: all hosts consume maximum power all the time. |
  | RandomConstants | If you are using any algorithms, policies or workload included in the power package please cite the following paper: Anton Beloglazov, and Rajkumar Buyya, "Optimal Online Deterministic Algorithms and Adaptive Heuristics for Energy and Performance Efficient Dynamic Consolidation of Virtual Machines in Cloud Data Centers", Concurrency and Computation: Practice and Experience (CCPE), Volume 24, Issue 13, Pages: 1397-1420, John Wiley & Sons, Ltd, New York, USA, 2012 |
  | RandomHelper | The Helper class for the random workload. |
  | RandomRunner | The example runner for the random workload. |
  | ThrMc | A simulation of a heterogeneous power aware data center that applies the Static Threshold (THR) VM allocation policy and Maximum Correlation (MC) VM selection policy. |
  | ThrMmt | A simulation of a heterogeneous power aware data center that applies the Static Threshold (THR) VM allocation policy and Minimum Migration Time (MMT) VM selection policy. |
  | ThrMu | A simulation of a heterogeneous power aware data center that applies the Static Threshold (THR) VM allocation policy and Minimum Utilization (MU) VM selection policy. |
  | ThrRs | A simulation of a heterogeneous power aware data center that applies the Static Threshold (THR) VM allocation policy and Random Selection (RS) VM selection policy. |

- Overview
- Package
- Class
- Use
- Tree
- Deprecated
- Index
- Help

- Prev Package
- Next Package

- Frames
- No Frames

- All Classes
